# A Statistically Grounded and Physics-Aware Vision Framework for Detecting Barely Visible Impact Damage (BVID) in Heterogeneous Polymer-Matrix Composites

**DOI:** 10.3390/polym18101240

**Published:** 2026-05-19

**Authors:** Gönenç Duran

**Affiliations:** 1 Automotive Technology Program, Department of Motor Vehicles and Transportation, Vocational School, Mudanya University, Bursa 16960, Türkiye; gonenc.duran@mudanya.edu.tr; 2 Mechanics and Advanced Materials Research Group Laboratory, Automotive Engineering Department, Engineering Faculty, Bursa Uludağ University, Bursa 16059, Türkiye

**Keywords:** Barely Visible Impact Damage (BVID), heterogeneous composite materials, low-velocity impact, YOLO, vision-based damage detection

## Abstract

Barely Visible Impact Damage (BVID) in heterogeneous polymer-matrix composites remains difficult to detect because subtle damage signatures are often masked by complex architectures, hybrid textures, and overlapping failure morphologies. This study therefore presents an experimentally grounded, physics-aware, and statistically validated vision-based inspection framework rather than a purely detector-centered benchmarking exercise. Real post-impact images were obtained from controlled low-velocity impact experiments on 20 composite architectures and 60 physical specimens, yielding approximately 2000 images across laminated, hybrid, textile-reinforced, and sandwich structures. The dataset was organized using a specimen-disjoint splitting protocol to prevent leakage across training, validation, and test subsets. To improve robustness while preserving physical realism, a physically grounded Albumentations strategy was developed using only physically admissible transformations and explicit exclusion of non-physical operations that could distort damage morphology or surface continuity. Model development was further complemented by a hybrid hardware workflow in which cloud-based GPU training was combined with deployment-oriented inference profiling on resource-constrained edge-like hardware, thereby linking detection accuracy to practical industrial feasibility. In addition, model performance was evaluated under a standardized training budget and validated through repeated runs, Friedman significance testing, and Holm-corrected Wilcoxon signed-rank pairwise comparisons to ensure error-controlled interpretation of inter-model differences. Across the evaluated compact YOLO families, YOLO26s delivered the strongest overall performance, reaching 0.841 mAP@0.5, 0.586 ± 0.004 mAP@0.5:0.95, and an F1-score of 0.809, while YOLO11s achieved the highest precision and YOLO26n remained competitive in recall with nano-level compactness. Overall, the results show that experimentally generated heterogeneous composite data, morphology-preserving augmentation strategy development, leakage-aware dataset design, deployment-oriented computational profiling, and statistically grounded validation together provide a more robust and application-relevant basis for automated BVID detection in polymer-matrix composite structures.

## 1. Introduction

Heterogeneous composite materials are widely used in the aerospace, automotive, defense, and energy sectors due to their superior properties, such as high specific strength, corrosion resistance, and design flexibility [1,2,3]. However, these same structural advantages are accompanied by an extremely complex mechanical response in terms of damage initiation, propagation, and visibility [4,5,6]. In addition, the multi-layered and architecture-sensitive nature of these materials causes the damage arising under low-velocity or repeated impact conditions to exhibit a complex, multimodal character that often leaves only limited traces on the surface [2,4,5,7,8]. Under service conditions, low-velocity impact can significantly reduce structural safety and residual strength by triggering critical damage modes such as delamination and Barely Visible Impact Damage (BVID), even when only faint surface marks are present [7,8,9,10]. In particular, impact damage such as BVID and Visible Impact Damage (VID) may initiate severe internal damage modes despite leaving only limited or, in some cases, clearly visible surface indications [11]. Although such damage may be difficult to detect on the surface, it constitutes a critical safety concern because it can induce severe internal damage mechanisms, including delamination, matrix cracking, fiber breakage, crushing, and, in the final stage, penetration [2,3,4,11].

In general, the fundamental challenge of BVID is not limited to the mere detection of damage presence but also requires the reliable discrimination of its morphological characteristics and progression level [7,10,12]. In heterogeneous composite structures, even under the same nominal impact condition, the visual appearance of damage may vary substantially depending on parameters such as fiber type, weave/topology, stacking sequence, thickness, matrix system, and sandwich/laminate architecture [4,6,13]. Therefore, the impact response represents an architecture-dependent problem not only in terms of material strength, but also in terms of visual damage representation [5,8,14,15,16]. Especially under progressive or repeated impact conditions, damage may evolve from early-stage crack initiation to delamination, and subsequently to localized fracture and penetration [4,5,7]. These challenges become even more complex not only in conventional laminates, but also in sandwich structures, hybrid composites, and architecturally heterogeneous reinforced systems, because impact energy absorption and damage propagation strongly depend on core–face interaction, interlaminar bonding, and reinforcement type/weave geometry [5,8,14]. For this reason, the rapid and reliable detection of impact-induced damage arising either after manufacturing or during service has become a fundamental requirement for quality assurance and structural health monitoring applications [2,7,17].

Although conventional Non-Destructive Testing (NDT) and Non-Destructive Inspection (NDI) methods (e.g., ultrasonic testing, thermography, and eddy current inspection) can provide high sensitivity, they often struggle to meet the objective of continuous/online inspection in every scenario due to application cost, operator dependency, cycle time, and difficulties in integration into production lines [2,18,19]. In contrast, imaging-based inspection techniques such as active thermography and shearography have been reported to be effective in distinguishing defect/damage regions in damaged composite structures; moreover, rapid approaches such as laser-line scanning thermography offer capabilities for defect/damage localization and characterization [5,20,21]. Consistent with this, high-throughput computer vision and image-based approaches, combined with low-cost hardware, provide a scalable alternative for industrial inspection by enabling high repeatability [5,6,7]. In this context, it is evident that NDT/NDI approaches offer important advantages for the detection of BVID and related damage modes in composite structures [2,7,22,23]. Nevertheless, most of these methods require additional hardware, expert interpretation, time, and cost; in some cases, they reach practical limitations in field-scale, high-volume, or rapid inspection scenarios [7,22]. Although multimodal studies based on the combined evaluation of thermographic and ultrasonic data have produced reliable results for BVID characterization, a substantial portion of this literature remains limited in terms of automatic visual representation of damage and integration into real-time decision-support systems [7,10,22].

From a broader perspective, the growing demands of Industry 4.0 and autonomous maintenance processes (Maintenance 4.0) have highlighted the need for rapid and automated detection systems that operate independently of human intervention [24,25,26]. These constraints have directed researchers toward Deep Learning (DL)-based computer vision methods that automate feature extraction [1,23,27]. Although two-stage detectors such as Faster R-CNN and Mask R-CNN, used in earlier studies, achieved high accuracy, their computational burden constitutes a bottleneck for real-time use on industrial production lines [26,28,29].

Recently, in order to overcome operational constraints and establish the required balance between speed and accuracy, there has been a growing shift toward new approaches that perform classification and localization simultaneously without requiring the time-consuming independent region proposal stage [28,30]. Among these, the one-stage You Only Look Once (YOLO) architecture, developed to address the need for real-time detection, revolutionized the field by formulating object detection as a regression problem and redefining the trade-off between speed and accuracy [31,32,33]. In the literature, numerous studies have demonstrated the success of earlier-generation YOLO models in composite damage detection [11,19,34]. For example, YOLO architectures have been used to detect fiber breakage on Carbon Fiber-Reinforced Polymer (CFRP) surfaces, achieving highly successful results with high sensitivity [11,19,35]. In addition, YOLOv8 has been reported to capture small defects more effectively thanks to its improved Feature Pyramid Networks (FPN) [31,36]. However, the majority of these studies have focused on composite material scenarios in which the background texture is relatively homogeneous, such as single-fiber systems or flat metallic surfaces [11,19,28,37,38]. In contrast, in hybrid composites with heterogeneous woven architectures, the high visual similarity between the natural surface pattern of the material and the damage appearance (low inter-class variance) can dramatically increase the false-positive rate of standard YOLO models, thereby highlighting the need for more studies conducted under realistic scenarios [2,11,31,39,40,41].

In industrial applications, the YOLO family of algorithms has been widely adopted and continuously developed because of its strong speed–accuracy balance in real-time object detection [30,42]. The YOLOv7 architecture established a strong foundation for real-time detection performance through its “trainable bag-of-freebies” approach [31,43,44]. Subsequent Ultralytics releases diversified both architectural and training components, while YOLOv8 offered balanced performance through its anchor-free head design and improved building blocks [28,31,45]. More recent developments have introduced YOLO11 and the latest YOLO26 architectures, which aim to improve detection sensitivity while reducing the number of parameters. While YOLO11 maintains broad task support through improvements in accuracy and computational efficiency, the newest YOLO26 approach targets lower latency, easier integration, high energy efficiency, and reduced cost through edge-device-oriented design and end-to-end NMS-free inference [28,46,47,48]. Owing particularly to mechanisms such as Grouped Query Attention and enhanced Cross Stage Partial (CSP) modules, these next-generation models offer more robust contextual learning capability in complex backgrounds [48]. Although these models are known to demonstrate superior performance across various domains, their behavior in the “structural noise” environment created by heterogeneous composite materials has not yet been sufficiently investigated in the literature. In particular, the efficiency of the YOLO26-n (nano) and YOLO26-s (small) variants—both suitable for edge devices with limited computational resources—in detecting complex damage morphologies is of considerable importance for energy cost, deployment feasibility, and economically scalable solutions [34,47].

In this context, stated differently, a growing body of research has shown that deep learning-based image processing systems for visual damage detection in composite structures have emerged as a powerful alternative for the automatic identification of defects and damage in composite materials [4,19,23,39]. In particular, studies focusing on the classification and detection of BVID and impact-induced defects from thermographic or direct visual images have demonstrated that deep learning architectures can learn low-contrast damage traces with complex morphologies [7,35,49]. In addition, image-based studies relying on microstructural features, drilling-induced defects, or internal defect representations have further highlighted the growing importance of automated quality control in composite materials [4,11,18,39,50]. Nevertheless, a substantial portion of this literature has either been developed on single type/homogeneous composite datasets or remained limited to a small number of architectures and highly controlled laboratory scenarios [23,27,34].

Another highly critical issue is that data partitioning strategies and augmentation protocols are often not discussed sufficiently in existing artificial intelligence-based damage detection studies [24,51,52,53]. Different images obtained from the same specimen, variations in illumination or pose, or sequential impact stages may be randomly distributed across training and test sets, thereby creating data leakage and artificially inflating performance metrics. This problem is not unique to the field of composites; rather, it has been extensively documented across many domains, including image classification, biomedical imaging, EEG analysis, and broader data science workflows. When different views of the same physical sample are shared across dataset partitions, the model may learn specimen-dependent visual traces rather than achieving true generalization. For this reason, specimen-disjoint or true event-level partitioning should be regarded as a fundamental requirement for reliable benchmark design in composite damage detection [53,54,55,56,57].

Within this scope, another fundamental challenge in image-based composite damage detection is the limited availability of labeled, system-specific experimental data [51,58,59]. Although a large number of images and open datasets are available online, the visual signature of damage in composite structures depends strongly on system parameters such as material architecture (e.g., sandwich, textile-reinforced, UD, or hybrid structures), surface texture, resin–fiber contrast, impact severity, illumination, and camera geometry [44,60,61]. For this reason, a representation learned from one dataset often cannot be generalized to another system with the same level of reliability because of domain shift, and the model may create industrial risk in the field by producing false positives or false negatives [6,34,57]. Therefore, it is considered more rational from the perspective of industrial applicability to begin with system-specific learning and then generalize this knowledge in a controlled manner [27,58].

Another methodological problem in deep learning-based damage detection is that the model may fail to distinguish physically meaningful damage patterns from incidental visual cues specific to the dataset [29,62]. This risk becomes even greater under small-data conditions, where the model becomes more prone to overfitting by learning dataset-specific background or texture cues rather than physically meaningful damage characteristics [31,56,63]. Data augmentation is a widely used strategy to mitigate this issue; however, generic augmentation techniques may distort physically meaningful morphologies such as crack orientation, delamination boundary gradients, or the linearity of fiber breakage, thereby artificially biasing model decisions and weakening industrial reliability [62,64].

Therefore, the objective is not merely to increase the amount of data, but rather to develop a physics-aware data preparation strategy that can diversify experimentally generated data representing the system’s own damage phenomenology without compromising physical realism [7,34]. Indeed, studies on woven/textile-based composites have shown that defects should not be defined simply in terms of “presence/absence,” but rather systematically characterized according to their morphology and spatial distribution, and that such a classification logic is critical for automation [50]. Similarly, studies on impact damage detection have reported that damage in composite structures can be captured through nonlinear indicators/responses under dynamic excitation, thereby supporting the need for controlled impact scenarios and rigorous validation [10,65]. In this context, the small-object and multi-scale detection literature emphasizes that variations in scale, contrast, and context directly influence the success of modern detectors, demonstrating that, without controlled experimental generation and proper data preparation, it is difficult to obtain a model that “works everywhere” [27,29,62,66].

Nevertheless, because many studies in the literature have been reported on different datasets and under different training settings, consistent and reproducible comparisons on the same problem definition are required in order to distinguish the true contribution of YOLO architectures. In addition, reporting only average accuracy metrics in model comparisons is widely regarded as an important methodological limitation [54,67]. In deep learning models, factors such as training stochasticity, initial weights, data shuffling, Albumentations-based transformations, and the overall augmentation pipeline can substantially influence the results. Despite this, many studies still claim a “best model” based on a single run or infer architectural superiority without testing statistical significance [68,69]. The erroneous interpretation of classifier and detector comparison results, as well as the problem of multiple comparisons, has been explicitly discussed in the methodological literature [70,71]. Indeed, in recent object detection studies comparing numerous variants of the YOLO family, objectively isolating whether small, reported performance gains (e.g., in mAP) arise from training-environment efficiency factors or from genuine algorithmic superiority requires that these methodological challenges be explicitly addressed [72,73]. In this regard, particularly under controlled experimental conditions with a limited number of repetitions, nonparametric statistical frameworks such as the Friedman test and the Holm-corrected Wilcoxon test provide reliable tools for comparison [67,70,74].

To address the aforementioned methodological and application-oriented gaps, this study focuses on the image-based detection of BVID and related damage modes generated through a controlled experimental impact campaign in heterogeneous composite architectures. In this work, physically meaningful, repeatable, and progressive damage scenarios were produced across 20 different composite architectures using a Split-Hopkinson-like impact jaw setup. The resulting post-impact images were organized under a specimen-disjoint partitioning strategy to prevent data leakage; during the annotation stage, a practical multi-class damage taxonomy was adopted to preserve industrial interpretability and avoid excessively fragmented class structures. In this way, the complex visual damage patterns observed on heterogeneous composite surfaces were systematically represented from an application-oriented and high-throughput inspection perspective.

Within this framework, the nano and small variants of YOLOv8, YOLOv10, YOLO11, and the next-generation YOLO26 architectures were trained and evaluated under a fair and standardized benchmarking protocol, using the same input resolution, the same epoch budget, the same seed logic, and the physics-aware data augmentation strategy developed specifically for this study. For performance evaluation, mAP@0.5:0.95 was adopted as the primary metric, as it provides a stricter and more generalizable assessment in object detection; this was complemented by mAP@0.5, Precision, Recall, and F1-score. In addition, to interpret model differences not only based on mean scores but also by accounting for variability across training repetitions, the nonparametric Friedman test and the Holm-corrected Wilcoxon signed-rank test were employed. Accordingly, this study approaches BVID detection in heterogeneous composites not merely as a computer vision problem, but as a multi-layered research problem in which experimental control, data integrity, physical interpretability, statistical validation, and computational cost are evaluated together, with the aim of providing a more reliable, reproducible, and application-oriented reference framework for next-generation autonomous damage detection systems.

## 2. Materials and Methods

**Figure 1:**
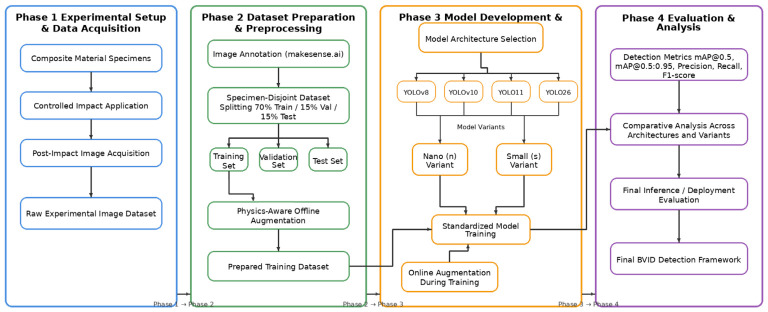
Overall workflow of the proposed experimental and vision-based framework for BVID detection in heterogeneous composite materials.

This section describes the experimental procedures used to generate impact-induced damage in heterogeneous composite materials and the vision-based detection methodology developed for Barely Visible Impact Damage (BVID) assessment using advanced YOLO architectures. As shown in Figure 1, the overall methodological pipeline of the study consists of four sequential phases: experimental setup and data acquisition, dataset preparation and preprocessing, model development and training, and performance evaluation and analysis. In this workflow, controlled impact loading was first applied to generate representative damage states, followed by image acquisition, annotation, specimen-disjoint dataset splitting, and augmentation-based data preparation. The resulting datasets were then used to train and benchmark YOLOv8, YOLOv10, YOLO11, and next-generation YOLO26 models in both nano (n) and small (s) variants under identical data splits, augmentation policies, and training hyperparameters, thereby ensuring strict fairness and statistical comparability across architectures.

### 2.1. Experimental Methodological Design of Progressive Impact Testing and High-Fidelity Data Acquisition

In this study, the experimental setup was developed to facilitate damage detection by employing a laboratory-scale impact framework that reproduces realistic damage progression in heterogeneous composites. Rather than relying on generic image datasets, the proposed workflow was grounded in a laboratory-scale impact campaign specifically developed to reproduce realistic damage initiation and progression under controlled loading conditions. In this way, the image dataset used for object detection was directly linked to experimentally generated damage states with clear mechanical relevance.

To generate representative impact-induced damage, controlled dynamic impact experiments were performed using a three-point bending configuration in accordance with ASTM D7250/D7250M-20 [75]. Under dynamic loading, composite materials exhibit deformation and failure responses that are strongly influenced by strain-rate sensitivity, reinforcement form, stacking sequence, and overall material architecture. As a result, the severity, spatial extent, and morphology of impact-induced damage may vary considerably across different composite systems, even when nominally identical loading conditions are applied. This architectural sensitivity is particularly important in heterogeneous composites, where local stiffness mismatch, interfacial incompatibility, and surface-texture variability may affect both damage evolution and its visual detectability [76,77].

**Figure 2:**
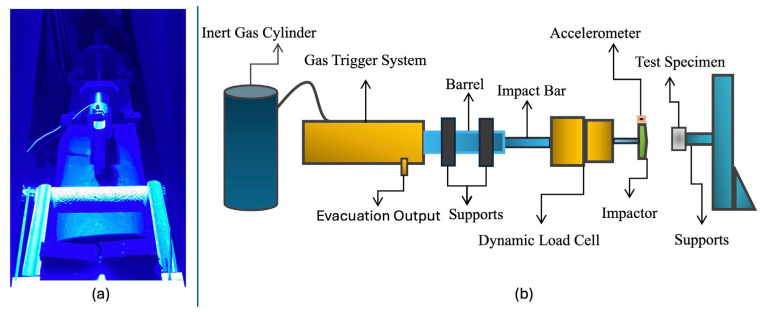
Front view (**a**) and schematic lateral view (**b**) of the impact test setup.

All impact experiments were conducted at the Applied Mechanics and Advanced Materials Research Group (UMİMAG) laboratory at Bursa Uludağ University using a YMC 9800 dynamic signal acquisition and analysis system. As illustrated schematically and visually in Figure 2, the experimental setup was based on a Split-Hopkinson-like impact jaw configuration adapted to apply localized transverse impact loading under three-point bending conditions. This configuration enabled repeatable impact delivery and controlled monitoring of damage formation under laboratory conditions [76,77,78].

The dynamic damage test rig consists of a gas-triggered loading system connected to a rod–load bar assembly. A dynamic load cell and an accelerometer were mounted on the load bar to measure impact force and acceleration during the loading event. At the distal end of the rod system, a loading head enables the application of a concentrated impact force to the specimen surface. Each composite specimen was positioned on two supports with a center span of 150 mm, forming a three-point bending configuration as shown in Figure 1.

For all dynamic experiments, the gas triggering system was pressurized to 10 bar, allowing an instantaneous dynamic load of approximately 250 kN to be applied to the specimen. Based on experimental measurements and calculations, the resulting strain rate was determined to be on the order of 10^2^ s^−1^. According to established classifications, the applied loading conditions can therefore be categorized as impact loading This Split-Hopkinson-like configuration ensures repeatable and controlled damage generation while preserving realistic impact conditions relevant to industrial composite components [77,78].

To reproduce the gradual accumulation of subcritical damage and the transition toward visually subtle but structurally meaningful BVID states, a progressive step-stress low-velocity impact (LVI) protocol was employed. In this protocol, the impact severity was increased incrementally from one loading stage to the next, allowing controlled observation of damage initiation, propagation, and eventual intensification. The loading sequence is defined as:

(1) $$S_{LVI}=E_{1},E_{2},\ldots ,E_{i},{\;E}_{1}<E_{2}<\cdots <E_{i}$$

where $S_{LVI}\;$ denotes the ordered impact loading sequence, *E_i* represents the impact energy applied at the *i*-th loading stage, and *i* is the total number of sequential impact steps imposed on a given specimen. The impact energy at each stage can be expressed as

(2) $$E_{i}=\frac{1}{2}m{v_{i}}^{2}$$

(3) $$E_{i+1}=E_{i}+\Delta E,\;\Delta E>0,i=1,2,\ldots ,i-1$$

where m  is the effective impactor mass and $v_{i}$ is the impact velocity corresponding to the i-th loading stage. Accordingly, each successive loading step satisfies $E_{i+1}>E_{i}$, ensuring a monotonic increase in impact severity throughout the experimental sequence. Here, ΔE denotes the prescribed energy increment between consecutive loading stages, which was selected to ensure gradual damage accumulation without causing immediate catastrophic failure in the early steps. This progressive design enabled the controlled generation of damage states ranging from incipient internal degradation to barely visible surface damage and, ultimately, to more pronounced failure modes. This formulation enabled the experimental protocol to capture the transition from subtle BVID states to more advanced and structurally significant damage conditions [79,80].

### 2.2. Composite Architectures

This study establishes a statistically grounded and physically consistent framework for the automatic detection of BVID in heterogeneous composite materials. Rather than relying on generic public datasets, the proposed methodology is based on controlled LVI experiments designed to reproduce real damage formation mechanisms. Progressive impact scenarios were generated using a Split-Hopkinson-like impact setup across 20 distinct composite architectures. The specimen pool comprised composite samples manufactured at the UMIMAG Laboratory of Bursa Uludağ University, as well as demonstrator materials sourced from various composite fairs. These architectures encompassed laminated, hybrid, textile-reinforced, and sandwich structures. To represent realistic structural variability, three replicated specimens were tested for each architecture, resulting in a total of 60 specimens. This progressive step-stress design enabled the capture of damage evolution from subtle surface indications to structurally significant BVID states. Following each impact event, the specimens were systematically imaged and annotated to construct the dataset used in this study. The composite architectures considered in the present study are outlined in Table 1.

### 2.3. Post-Impact Imaging Procedure and Experimental BVID Data Acquisition

Following each controlled low-velocity impact event, the composite specimens were systematically imaged to document visible and barely visible surface damage and to construct the experimental BVID dataset [81]. The imaging procedure was designed to capture realistic post-impact damage patterns across heterogeneous composite architectures under consistent inspection conditions [60]. The acquired images included damage features such as delamination boundaries, local interfacial debonding, matrix crack traces, fiber fracture lines, fiber bundle disruption, indentation zones, surface crushing, puncture marks, and, where present, perforation-related material loss [5,7]. The resulting images were subsequently annotated manually using makesense.ai, a browser-based online annotation platform, where bounding-box labels were assigned to define damage regions for object-detection training. For industrial interpretability and to reduce excessive overlap among overly fragmented labels, these morphologies were grouped into four practical categories: delamination/interfacial damage, matrix cracking, fiber breakage, and penetration/perforation [39,82]. In this way, the dataset was derived directly from experimentally observed post-impact damage regions spanning subtle BVID indications to more severe impact-induced damage states [60,81].

### 2.4. Imaging Procedure and Acquisition Conditions

To ensure that the learned visual features correspond to actual damage rather than acquisition artifacts, all images were captured under controlled and repeatable conditions. The camera position, working distance, lighting configuration, and exposure-related settings were kept fixed throughout the campaign.

The acquired image set can be written as

(4) $$X={\left\{x_{i}\right\}}_{i=1}^{N}$$

where

X denotes the full image collection;
$x_{i}$ is the i-th acquired image;
N  is the total number of images.

This controlled acquisition strategy minimizes unwanted variability arising from illumination fluctuations, surface reflections, and geometric inconsistency, thereby enhancing the reliability and reproducibility of the downstream learning process. The detailed imaging setup and acquisition conditions are presented in Table 2.

### 2.5. Creation of Defect Taxonomy and Classification of Visual Characteristics

To balance physical interpretability and machine learning feasibility, the observed damage patterns were grouped into a four-class taxonomy:

(5) $$C=\{c_{1},c_{2},c_{3},c_{4}\}\;$$

where

C: All class taxonomy;
$c_{1}$: delamination/interfacial damage;
$c_{2}$: matrix cracking;
$c_{3}$: fiber breakage;
$c_{4}$: penetration/perforation.

Each image was annotated using bounding boxes, yielding the labeled dataset

(6) $$D=\{{(x}_{i},Y_{i}){\}}_{i=1}^{N}$$

where

D is the full labeled object-detection dataset;
$x_{i}$ is the *i*-th image;
$Y_{i}$ is the annotation set associated with image *x_i*;
N  is the number of labeled images.

For each image ${\;x}_{i}$, the annotation set is defined as

(7) $$Y_{i}={\left\{(b_{ij},c_{ij})\right\}}_{j=1}^{M_{i}}$$

where

$b_{ij}\;$ is the bounding box of the *j*-th damage instance in image i;
$c_{ij}\in C$ is the class label assigned to that instance;
$M_{i}\;$ is the number of annotated defect instances in image i.

This formulation enables the simultaneous localization and classification of defects in a manner fully consistent with the object-detection framework adopted in this study. The detailed damage taxonomy and annotation protocol are provided in Table 3.

### 2.6. Dataset Composition and Specimen-Disjoint Splitting Strategy

A critical methodological concern in image-based defect detection is data leakage, particularly when multiple images derived from the same physical specimen are distributed across training and testing subsets [34]. To mitigate this risk, the dataset was established from experimentally generated impact damage, with post-impact images acquired under controlled inspection conditions. The experimental dataset was subsequently expanded with controlled synthetic augmentations to enhance sample diversity while preserving physical realism, yielding a final dataset of approximately 2000 images. Crucially, the training, validation, and test sets were partitioned at the specimen level, ensuring that all original images and their augmented derivatives associated with a given specimen remained within the same subset. This specimen-disjoint strategy reduced leakage risk limited the possibility of specimen-specific texture memorization, and provided a more rigorous assessment of model generalization to previously unseen composite specimens. To prevent this, a specimen-disjoint split was enforced.

Let the specimen set be:

(8) $$S=\{s_{1},s_{2},\ldots ,s_{M}\}\;$$

where

- S is the set of all physical specimens;
- $s_{k}$ is the k-th specimen;
- M is the number of specimens.

The partitioning condition is defined as

(9) $$S_{train}\cap S_{val}=\emptyset \;S_{train}\cap S_{test}=\emptyset \;\;S_{val}\cap S_{test}=\emptyset$$

where

- $S_{train}$ is the training specimen subset;
- $S_{val}$ is the validation specimen subset;
- $S_{test}$ is the test specimen subset;
- ∅ indicates the empty set.

Complete coverage is ensured by:

(10) $$S_{train}\cup S_{val}\cup S_{test}=S$$

meaning that all specimens are included in exactly one subset.

This partitioning strategy prevents the detector from exploiting specimen-specific surface signatures, thereby enforcing genuine generalization to previously unseen specimens. The detailed dataset composition and specimen-disjoint splitting strategy are provided in Table 4.

### 2.7. Physics-Aware Augmentation Strategy and Ablation Design

Owing to the limited size and experimentally constrained variability of the acquired BVID dataset, a physics-aware augmentation strategy was implemented to improve generalization performance while maintaining morphological fidelity. Rather than relying on unconstrained generic augmentations, the adopted pipeline was restricted to transformations that remained consistent with composite damage mechanisms, surface texture, and inspection physics.

An augmented sample is defined as

(11) $$\tilde{x}=T\left(x\right),\;T\in T_{phys}$$

where

- x is the original image;
- $\tilde{x}$ is the augmented image;
- T(⋅) is the augmentation operator;
- $T_{phys}$ is the set of physically valid transformations.

To conceptually constrain domain shift, the augmented distribution was required to remain close to the real distribution:

(12) $$D_{KL}\left(P_{real}\;|\;P_{aug}\right)\ll 1$$

where

- $D_{KL}(\cdot ∥\cdot )$ is the Kullback–Leibler divergence;
- $P_{real}$ is the real-image distribution;
- $P_{aug}$ is the augmented-image distribution.

Although this divergence was not explicitly optimized during training, it serves as the conceptual basis for limiting augmentation intensity and avoiding unrealistic transformations. The ablation study used three dataset configurations:

(13) $$D_{raw},\;{\;D}_{phys},\;{\;D}_{hyb}\;$$

where

- $D_{raw}$ is the raw experimental dataset;
- $D_{phys}$ is the physics-aware augmented dataset;
- $D_{hyb}$ is the hybrid dataset formed by combining physics-aware samples with curated external but domain-aligned data.

Although this divergence was not explicitly optimized during training, it provided the conceptual basis for constraining augmentation intensity and excluding unrealistic transformations. Accordingly, the ablation study was structured around three dataset configurations, namely the raw experimental dataset ($D_{raw}$), the physics-aware augmented dataset ($D_{phys}$), and the hybrid dataset combining physics-aware samples with curated external yet domain-aligned data ($D_{hyb}$). The dataset strategy adopted for this controlled ablation design is summarized in Table 5.

To preserve morphological fidelity and maintain alignment with real inspection conditions, the augmentation policy was bounded by explicit physical and domain-consistency constraints. These physics-aware augmentation rules and the associated domain consistency framework are detailed in Table 6.

### 2.8. YOLO Architectures and Standardized Training Protocol

To define a realistic benchmark for autonomous inspection systems with constrained computational capacity, the nano and small variants of successive YOLO generations were evaluated under a strictly standardized protocol. The investigated architectures comprised YOLOv8, YOLOv10, YOLO11, and YOLO26, thereby spanning established anchor-free baselines, efficiency-oriented NMS-free detectors, attention-enhanced variants, and a next-generation end-to-end edge-oriented model [37,60,83].

YOLOv8 was adopted as a reference architecture because of its anchor-free head design and decoupled detection structure. YOLOv10 was included for its NMS-free formulation and dual label-assignment mechanism, both aimed at lowering inference latency. YOLO11 extended this comparison by incorporating C3k2 bottleneck blocks and C2PSA spatial attention, which are particularly relevant for suppressing background complexity in heterogeneous composite surfaces. YOLO26 was investigated as the next-generation model of the study due to its end-to-end NMS-free prediction scheme, DFL-free formulation, STAL-based small-target sensitivity, ProgLoss-driven training balance, and MuSGD optimization strategy. These properties were considered especially relevant for detecting subtle impact-induced micro-defects embedded in visually noisy composite textures [28,47,60].

For strict fairness, all models were trained using identical settings: an input resolution of 640 × 640, a fixed training budget of 300 epochs, COCO-pretrained weights, and three independent repetitions under controlled random seed conditions. Early stopping was intentionally disabled to avoid introducing convergence-related bias. Accordingly, the benchmark was designed to isolate the effect of architectural differences while preserving reproducibility and comparability across all model variants. And also, the benchmark included successive YOLO generations in nano (n) and small (s) variants:

(14) M={YOLOv8n,YOLOv8s,YOLOv10n,YOLOv10s,YOLOv11n,YOLOv11s,YOLO26n,YOLO26s}

where M denotes the model set used in the study.

For each model m∈M, the training objective is written as

(15) $$\theta_{m}^{*}=\arg\underset{\theta }{\min}L_{m}\left(\theta ;D_{train}\right)$$

where

- θ denotes the trainable parameter set;
- $\theta_{m}^{*}$ is the optimized parameter set for model m;
- $L_{m}$ is the loss function associated with model m;
- $D_{train}$ is the training dataset.

To ensure strict fairness and architectural comparability, all models were trained under the same input resolution, optimization budget, initialization policy, and evaluation framework. Table 7 summarizes the evaluated YOLO architectures in terms of their benchmark positioning, defining characteristics, model complexity profiles (parameter count and GFLOPs), and deployment-oriented platform configuration for cloud-to-edge computational assessment.

To ensure that architectural comparisons were not confounded by training-related variability, all models were trained under a strictly standardized protocol. In addition, practical repeatability was assessed through three independent runs with different random seeds, and the resulting performance was reported in terms of mean ± standard deviation. The complete standardized training configuration, together with the compute fairness and reproducibility framework adopted in this study, is summarized in Table 8.

### 2.9. Hybrid Hardware Environment and Deployment-Oriented Computational Profiling

To assess the industrial deployability of the proposed models, training and inference were evaluated within a hybrid computational environment comprising both cloud and edge hardware. Training was conducted on the Google Colab cloud platform (Google LLC, Mountain View, CA, USA) using an NVIDIA A100-SXM4-40GB GPU (NVIDIA Corporation, Santa Clara, CA, USA). Inference and real-time performance testing were conducted on an Apple Mac mini M4 workstation (Apple Inc., Cupertino, CA, USA) equipped with a 10-core CPU, 10-core GPU, and 16-core Neural Engine, using the Metal Performance Shaders (MPS) backend. This hybrid setup enabled the joint evaluation of high-performance model training and edge-level deployment feasibility under practical hardware conditions [29,84,85].

To quantify the computational cost of the evaluated architectures, several deployment-relevant metrics were reported. These included the number of model parameters, representing storage and memory burden; GFLOPs, reflecting computational complexity; inference latency per image on both the Mac mini M4 (MPS) and the Colab NVIDIA GPU environments; and frames per second (FPS), indicating real-time processing capability. Together, these metrics provide a deployment-oriented basis for comparing model efficiency alongside detection performance [29,31,85,86].

Let $t_{inf}$ denote the mean inference latency per image in milliseconds. The frame rate is then defined as

(16) $$FPS=\frac{1000}{t_{inf}}$$

where

- FPS is frames per second;
- $t_{inf}$ is inference latency in milliseconds per image.

This relation provides a standardized basis for comparing deployment-oriented efficiency across models under different computational environments. The corresponding hybrid hardware and computational environment are presented in Table 9.

### 2.10. Quantitative Evaluation Metrics and Confidence Threshold Selection

Detection performance was assessed using standard object-detection metrics. The overlap between a predicted bounding box *B_pand* a ground-truth box *B_gis* measured using Intersection-over-Union (*IoU*) [87,88]:

(17) $$IoU=\frac{\left|B_{p}\cap B_{g}\right|}{\left|B_{p}\cup B_{g}\right|}$$

where

- $B_{p}$ is the predicted bounding box;
- $B_{g}$ is the ground-truth bounding box;
- $|B_{p}\cap B_{g}|$ is the intersection area;
- $|B_{p}\cup B_{g}|$ is the union area.

From true positives (TP), false positives (FP), and false negatives (FN), the following metrics are computed:

(18) $$Precision=\frac{TP}{TP+FP}$$

(19) $$Recall=\frac{TP}{TP+FN}$$

(20) $$F1=2\cdot \frac{Precision\cdot Recall}{Precision+Recall}$$

where

- TP is the number of correctly detected objects;
- FP is the number of incorrect detections;
- FN is the number of missed ground-truth objects [34,89].

Model performance was assessed through both qualitative and quantitative analyses. Qualitatively, the predicted bounding boxes and confidence scores were examined to determine whether the models localized impact-induced damage in a visually meaningful and physically interpretable manner. Particular attention was paid to subtle defect patterns, since barely visible damage in heterogeneous composite surfaces may produce weaker visual cues and therefore lower confidence levels. This visual assessment helped identify not only successful detections, but also systematic failure modes such as missed detections, ambiguous boundaries, and confusion between morphologically similar damage categories [34,90].

Quantitatively, performance was evaluated using standard object-detection metrics derived from the confusion matrix, including Precision, Recall, F1-score, and mAP@0.5. These metrics were used to measure detection reliability, sensitivity to actual damage, and the overall balance between false alarms and missed defects. In addition to accuracy-based criteria, inference time and frames per second were also considered to assess deployment feasibility in real-time or near-real-time inspection scenarios. To complement the numerical results, representative true-positive, false-positive, and false-negative examples were visually analyzed, allowing the practical decision behavior of the models to be interpreted under realistic manufacturing conditions.

### 2.11. Statistical Validation and Error-Controlled Multiple-Comparison Protocol

To determine whether the observed performance differences reflected genuine architectural effects rather than random variation across repeated runs, model comparisons were conducted within a two-stage nonparametric framework. First, the Friedman test was used as an omnibus procedure to assess whether statistically significant differences existed among the evaluated models at the group level. This choice is consistent with established recommendations for comparative evaluation of multiple learning algorithms under repeated experimental settings [67].

When the omnibus null hypothesis was rejected, post hoc pairwise comparisons were performed using the Wilcoxon signed-rank test, a rank-based procedure originally developed for paired comparisons and widely used when distributional assumptions are undesirable or difficult to justify. To avoid inflated Type, I error under multiple pairwise testing, the resulting *p*-values were adjusted using Holm’s sequentially rejective procedure, which provides strong control of the family-wise error rate while remaining less conservative than the classical Bonferroni correction. Accordingly, the adopted protocol provided a statistically rigorous and reproducible basis for significance testing, pairwise model discrimination, and final performance ranking [91]. Within this formulation, let $y_{m,r}$ denote the performance score obtained by model *m* in repetition r, where m indexes the evaluated architecture and *r* indexes the independent training repetition. The global null hypothesis for the Friedman test was defined as

(21) $$H_{0}:\mu_{1}=\mu_{2}=\ldots \;=\mu_{K}$$

where

- $\mu_{k}$ is the central tendency of the k-th model’s performance;
- K is the number of compared models.

This hypothesis was tested using the Friedman test. If rejected, pairwise comparisons were conducted using the Wilcoxon signed-rank test with Holm correction:

(22) $$H_{0}^{\left(i,j,\ldots \right)}:\mu_{i}=\mu_{j}$$

where

- $\mu_{i}$ and $\mu_{j}$ are the performances of models i and j, respectively.

Nonparametric tests were selected because model performance across repeated runs does not necessarily satisfy Gaussian assumptions [67,70,74,91].

The statistical testing framework adopted for the rigorous comparison of the evaluated YOLO architectures is summarized in Table 10. The methodology combines a nonparametric Friedman test for global differences with Holm-corrected Wilcoxon signed-rank tests for pairwise comparisons, ensuring both statistical validity and control of family-wise error rates.

### 2.12. Methodological Positioning

The contribution of this study is not the proposal of a new backbone architecture, but the establishment of a reproducible, physically grounded, leakage-resistant, and statistically validated evaluation framework for BVID detection in heterogeneous composites. By integrating controlled experiments, specimen-disjoint splitting, physics-aware augmentation, standardized benchmarking, and nonparametric statistical validation, the methodology provides a reliable basis for comparing next-generation object detectors under realistic industrial inspection conditions.

Model performance was evaluated using standard object detection metrics:

- mAP@0.5;
- mAP@0.5:0.95 (primary metric);
- Precision;
- Recall;
- F1-score.

To ensure that observed performance differences are statistically reliable and not due to random variation, a rigorous statistical validation framework was implemented.

First, model performances obtained from repeated runs were analyzed using the nonparametric Friedman test, which is suitable for comparing multiple models under identical experimental conditions. Upon detecting a statistically significant overall difference (*p* < 0.05), pairwise comparisons were conducted using the Holm-corrected Wilcoxon signed-rank test to control the family-wise error rate.

This two-stage statistical approach ensures that performance claims are both robust and scientifically grounded.

## 3. Results and Discussion

### 3.1. Overall Detection Performance Across YOLO Architectures

**Figure 3:**
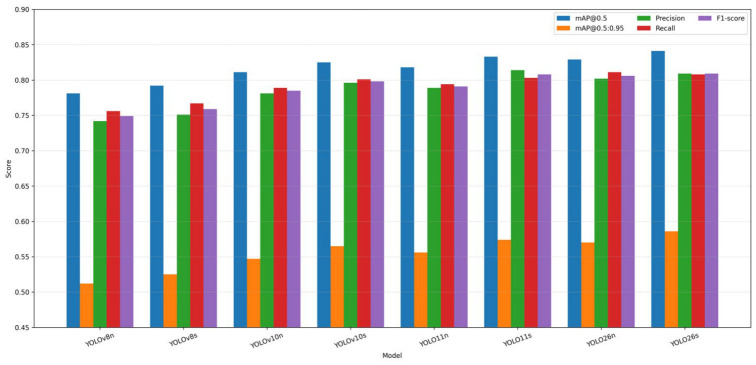
Comparative metric profiles of the evaluated YOLO architectures across mAP@0.5, mAP@0.5:0.95, Precision, Recall, and F1-score.

The overall comparative results revealed a structured but partially overlapping performance hierarchy across the evaluated YOLO architectures. As shown in Figure 3 and summarized quantitatively in Table 11, the strongest models were generally concentrated within the most recent compact next-generation variants, whereas the earlier baseline architectures remained at the lower end of the ranking. This pattern indicates that the investigated BVID detection task, despite being performed on experimentally grounded and heterogeneous composite surfaces, was sufficiently sensitive to expose meaningful differences among the successive YOLO generations.

Among all evaluated models, YOLO26s produced the strongest overall detection profile. It achieved the highest values for mAP@0.5 (0.841 ± 0.004), mAP@0.5:0.95 (0.586 ± 0.004), and F1-score (0.809 ± 0.005), indicating the most favorable balance between localization quality and decision consistency. This result suggests that the architectural refinements of the latest generation translated into a measurable advantage not only in coarse object-level detection, but also in stricter localization-sensitive performance. Since mAP@0.5:0.95 penalizes partial or imprecise bounding-box placement more strongly than mAP@0.5, the superiority of YOLO26s under both criteria indicates that its benefit extends beyond simple object presence recognition toward more spatially stable damage localization [31,88].

The remaining upper-tier architectures displayed more specialized strengths. YOLO11s achieved the highest Precision (0.814 ± 0.006), indicating comparatively stronger suppression of false-positive activations in visually ambiguous regions. This is particularly relevant for heterogeneous composite surfaces, where intact reinforcement texture, resin-rich zones, or architecture-dependent visual transitions may resemble real damage patterns. In contrast, YOLO26n yielded the highest Recall (0.811 ± 0.009), suggesting improved sensitivity to subtle or weakly expressed BVID instances while preserving nano-level model compactness. Accordingly, the leading models did not dominate uniformly across all metrics; instead, they exhibited different operational tendencies depending on whether balanced detection, conservative false-positive control, or sensitivity to weak damage cues was emphasized [37,48].

A clear separation was also observed between the weakest baseline models and the best-performing next-generation variants. YOLOv8n produced the lowest results across most of the principal metrics, reaching 0.781 ± 0.01 mAP@0.5, 0.512 ± 0.008 mAP@0.5:0.95, 0.742 ± 0.01 Precision, and 0.749 ± 0.01 F1-score. Although YOLOv8s provided a modest improvement over its nano counterpart, both baseline variants remained below the strongest compact models across all major criteria. This finding indicates that, within the present BVID inspection context, earlier lightweight baselines were less capable of resolving the combined challenges of subtle damage morphology, low-contrast boundaries, and heterogeneous background structure.

At the same time, the ranking within the mid-to-upper performance band was less sharply separated. Models such as YOLOv10s, YOLO11n, YOLO11s, and YOLO26n remained relatively close across several metrics, with partially overlapping mean ± std ranges. This suggests that, although the overall hierarchy was clear at the extremes, the intermediate compact models formed a more competitive cluster in which numerical differences alone should be interpreted cautiously. In practical terms, the results imply that the main advantage of the best-performing architectures lies not in an isolated gain in a single metric, but in their ability to maintain a more stable and balanced detection profile across localization-sensitive, precision-sensitive, and composite evaluation criteria.

Overall, the results presented in Figure 3 and Table 11 show that YOLO26s provides the strongest composite performance across the primary detection metrics, while YOLO11s and YOLO26n emerge as strong alternative candidates when precision-oriented and recall-oriented behavior are prioritized, respectively. These findings establish the general performance hierarchy of the benchmark and provide the basis for the more detailed metric-wise, qualitative, and statistical analyses presented in the following subsections.

### 3.2. Metric-Wise and Class-Wise Detection Behavior

Although the overall ranking presented in Figure 3 and Table 11 provides a useful first-level comparison, the behavior of the evaluated architectures becomes more informative when the results are interpreted at both the metric and class levels. In particular, the contrast between mAP@0.5 and mAP@0.5:0.95 reveals whether a model’s apparent success is driven mainly by coarse object-level detection or by more precise and spatially consistent localization. Under the present BVID inspection scenario, this distinction is especially important because impact-induced damage regions are often visually weak, spatially diffuse, or only partially bounded by sharp edges. Consequently, models that remain strong under the stricter mAP@0.5:0.95 criterion can be considered more reliable in localizing subtle damage morphology rather than merely recognizing its approximate presence.

From this perspective, the performance profile of YOLO26s is particularly noteworthy. Its leading performance in both mAP@0.5 and mAP@0.5:0.95 indicates that the model not only identifies damage-bearing regions with high confidence but also preserves stronger spatial agreement with the annotated ground-truth boxes. This suggests that the architectural refinements embedded in YOLO26s improve the consistency of box regression under heterogeneous composite surface conditions, where the visual transition between damaged and undamaged material is often gradual rather than sharply delimited. By contrast, several mid-tier models remain competitive at mAP@0.5 but show a narrower advantage under mAP@0.5:0.95, indicating that part of their apparent success may be associated with approximate region recognition rather than highly accurate localization.

The relationship between Precision and Recall further highlights the trade-off between conservative and sensitive detection behavior. YOLO11s, which achieved the highest Precision, appears to be the most conservative architecture among the upper-tier models. This suggests stronger control over false-positive responses, meaning that the model is less likely to misinterpret intact but visually textured regions as damage. Such behavior is beneficial in heterogeneous composites because woven surface patterns, hybrid reinforcement interfaces, or local reflectance fluctuations may generate damage-like visual cues even in the absence of real impact-induced defects. In contrast, YOLO26n, which attained the highest Recall, appears to prioritize sensitivity to weak damage cues more strongly. This makes it particularly effective in minimizing missed detections, even if that sensitivity may come at the cost of slightly less conservative decision behavior. The leading F1-score of YOLO26s confirms that its superiority is not driven by an isolated advantage in either conservatism or sensitivity alone, but rather by a more stable balance between false-positive suppression and true damage preservation.

**Figure 4:**
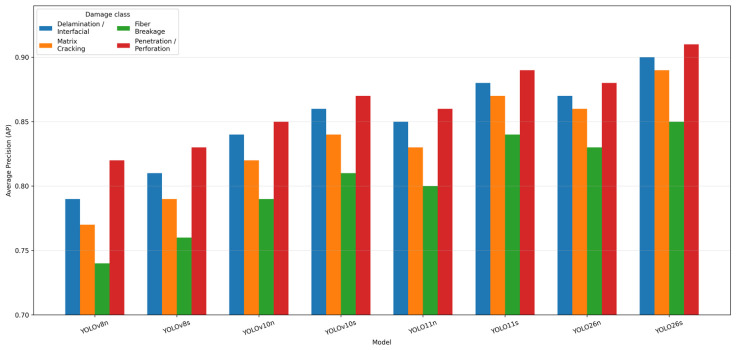
Per-class AP comparison across the proposed BVID taxonomy.

A more detailed view of model behavior emerges from the class-wise AP comparison shown in Figure 4. The results indicate that the benchmark hierarchy is not uniform across the proposed BVID taxonomy. Damage classes with more spatially coherent and visually distinguishable manifestations, such as penetration/perforation and delamination/interfacial damage, tended to achieve higher AP values across most architectures. In contrast, fiber breakage and, to a lesser extent, matrix cracking remained more challenging, especially in visually heterogeneous backgrounds where crack-like linear patterns, local texture interruptions, and material-intrinsic surface irregularities could partially mimic true damage morphology [50,61]. This class-dependent separation suggests that the observed model differences cannot be explained solely by overall architecture quality but are also conditioned by the visual discriminability of the target damage class itself.

Within this class-wise structure, YOLO26s again showed the most favorable overall profile by remaining consistently strong across all damage categories rather than achieving high performance in only one or two classes. YOLO11s also exhibited competitive class-level behavior, particularly in classes where false-positive suppression is important, whereas YOLO26n remained attractive in classes involving weak or partially diffuse damage expression. These tendencies are consistent with the global metric results: models that favor precision tend to behave more conservatively in ambiguous categories, whereas recall-oriented models are more responsive to subtle but uncertain damage traces. Accordingly, the class-wise AP analysis complements the metric-level trade-off interpretation by showing how each detector distributes its strengths and limitations across the actual defect taxonomy.

**Figure 5:**
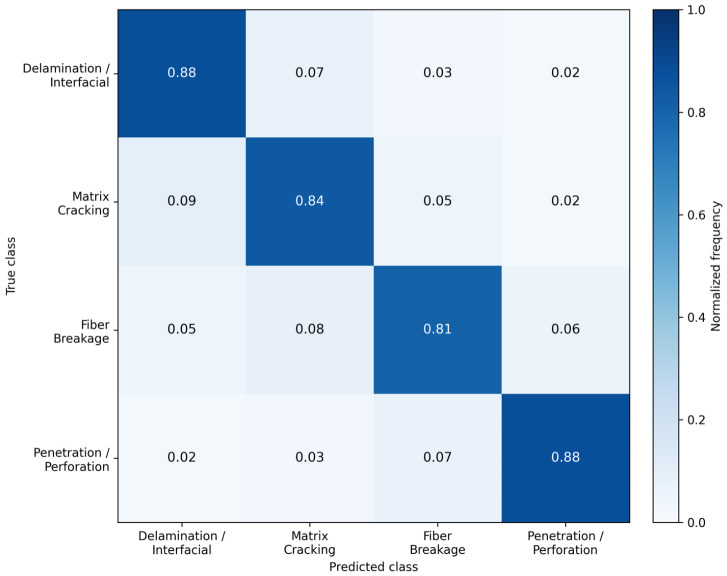
Class-wise confusion matrix of the best-performing YOLO architecture (YOLO26s).

The confusion matrix of the best-performing model, presented in Figure 5, further clarifies the source of residual errors at the class level. Although the diagonal dominance confirms that the detector separates the four proposed damage categories with generally high reliability, the off-diagonal entries reveal that the remaining errors are not random. Instead, they are concentrated between visually or morphologically adjacent classes. Confusion between delamination/interfacial damage and matrix cracking, as well as between fiber breakage and the neighboring crack-related categories, suggests that the detector occasionally struggles when the damage boundary is diffuse, the failure morphology is mixed, or the visible surface manifestation reflects more than one underlying damage mechanism. This is physically plausible in heterogeneous composite systems, where local impact response may not produce a single clean failure signature.

From an interpretive standpoint, the confusion structure shown in Figure 4 is especially valuable because it links the class-wise AP behavior to a physically meaningful error pattern. Classes with lower AP are also those more prone to mutual confusion, indicating that reduced class performance is associated not merely with weaker confidence, but with genuine ambiguity in visual separability. This supports the methodological choice of adopting a practical four-class taxonomy rather than a more fragmented label space, since excessive subdivision would likely amplify class overlap and reduce annotation consistency without necessarily improving industrial interpretability.

Overall, the combined evidence from Figure 3, Figure 4 and Figure 5 indicates that the strongest-performing architectures differ not only in absolute score level, but also in how effectively they preserve localization quality, balance precision against recall, and separate the proposed damage classes under heterogeneous visual conditions. The benchmark is therefore not governed by a single monotonic ranking, but by a multidimensional trade-off structure in which different models occupy different positions on the localization–precision–recall–class-separability continuum. This observation provides an important transition from raw metric comparison to the more detailed statistical validation presented in the following subsection.

### 3.3. Qualitative Detection Examples Across Heterogeneous Composite Surfaces

To complement the metric-level and class-wise quantitative comparisons, a broad set of representative true-positive detection examples was examined across heterogeneous composite materials with varying surface textures, reinforcement architectures, and damage morphologies. Because BVID and related impact-induced damage do not always appear as sharply bounded or high-contrast defects, but often emerge as weak, diffuse, or morphology-dependent surface disturbances embedded in structurally complex backgrounds, qualitative inspection remains important for verifying whether the detector response is physically meaningful rather than merely numerically competitive.

The representative examples collected for this study show that true-positive detections were obtained across all four proposed damage categories, namely delamination/interfacial damage, matrix cracking, fiber breakage, and penetration/perforation. A key observation is that the visual manifestation of these classes varies substantially across composite architectures. In some cases, damage appears as an elongated disturbed region with relatively coherent boundaries, whereas in others it is expressed through fragmented crack-like traces, localized fiber-disruption patterns, or compact perforation-centered zones. Despite this variability, the detector was generally able to localize the physically relevant damage region with acceptable spatial consistency.

**Figure 6:**
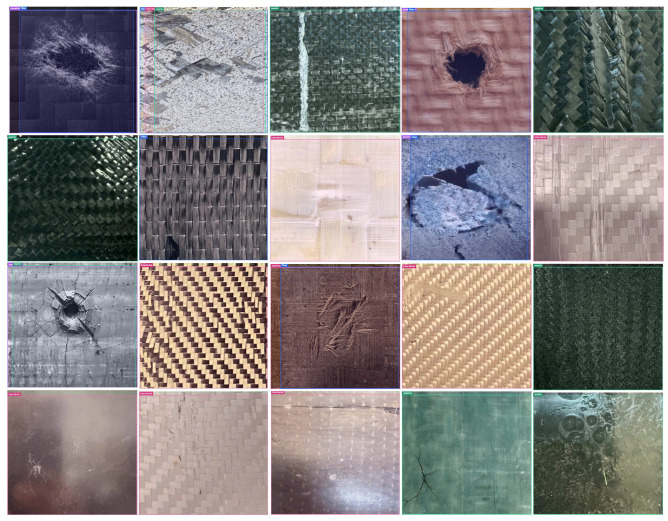
Representative true-positive examples identified for each defect taxonomy.

As illustrated by the react examples shown in Figure 6, successful detections were not restricted to a single material family or surface type but extended across visually distinct composite architectures. This suggests that the learned representation was not tied to a single texture-specific appearance but instead retained a broader degree of robustness under heterogeneous inspection conditions. The examples presented in Figure 6 further indicate that the most visually distinctive categories, particularly penetration/perforation and several interfacial-type damage cases, tend to yield more spatially coherent detections, whereas matrix cracking and fiber breakage may still exhibit greater intra-class variation even when correctly detected.

To formalize the interpretation of these representative true-positive examples, the dominant qualitative patterns observed across the figure are summarized in Table 12. Rather than listing isolated image-specific comments, the table organizes the main interpretive dimensions of the qualitative analysis, including cross-category coverage, intra-class variability, cross-architecture robustness, localization plausibility, and alignment with the quantitative class-wise trends.

### 3.4. Statistical Validation of Comparative Model Performance

To determine whether the observed metric differences reflected genuine architectural effects rather than stochastic variation across repeated runs, the comparative results were further examined using a two-stage nonparametric statistical framework. This step was necessary because the mean ± std trends reported in Table 11 and the metric-wise trade-offs discussed in the previous subsections, although informative, do not by themselves establish whether the ranking pattern remains statistically reliable under repeated training conditions. In compact-model benchmarking, where several architectures may differ only marginally in central metric values, statistical validation is essential to distinguish between robust separation and numerical overlap.

The omnibus statistical comparison results are summarized in Table 13. Across the five primary detection metrics, the Friedman test revealed statistically significant overall differences for mAP@0.5, mAP@0.5:0.95, Precision, and F1-score, whereas Recall remained non-significant or only borderline significant. This pattern is fully consistent with the performance structure observed in Table 12. In particular, the significant Friedman outcomes for the mAP-based metrics and F1-score indicate that the benchmark hierarchy is most clearly expressed in terms of localization-sensitive and balanced decision behavior. By contrast, the non-significant result for Recall suggests that the upper-tier models remain more compressed and partially overlapping in terms of sensitivity alone, which reduces the strength of global rank separation across repeated runs [67,70,71].

A second important point emerging from Table 13 concerns the best average-rank model associated with each metric. YOLO26s occupies the most favorable average-rank position for mAP@0.5, mAP@0.5:0.95, and F1-score, confirming that its superiority is not limited to a single raw mean value but is sustained across repeated comparisons. In contrast, YOLO11s appears as the best average-rank model for Precision, whereas YOLO26n occupies the same position for Recall. This ranking structure supports the interpretation already suggested by the metric-wise analysis: the strongest models differ not only in magnitude of performance, but also in the particular detection tendency they optimize, whether toward balanced composite behavior, conservative false-positive control, or recall-oriented sensitivity.

However, the omnibus results in Table 14 do not identify which model pairs are separated in a statistically reliable manner. For that reason, the post hoc pairwise comparison results based on Holm-corrected Wilcoxon signed-rank testing are presented in Table 15. These results show that the strongest statistically reliable separations were concentrated between the best-performing next-generation architectures and the weakest baseline variants. This pattern is particularly evident for mAP@0.5, mAP@0.5:0.95, and F1-score, where comparisons such as YOLO26s vs. YOLOv8n, YOLO26s vs. YOLOv8s, and YOLO11s vs. YOLOv8n remained significant after family-wise error correction. These outcomes indicate that the superiority of the strongest models over the weakest baselines is not merely descriptive, but sufficiently stable across repeated runs to remain statistically supported after multiplicity adjustment [67,70,71].

At the same time, Table 14 also highlights an equally important negative result: a substantial number of comparisons within the mid-to-upper group remained non-significant after Holm correction. Model pairs such as YOLO26s vs. YOLO11s, YOLO26n vs. YOLO11s, and YOLOv10s vs. YOLO11n repeatedly failed to reach adjusted significance despite visible numerical differences in Table 13. This indicates that several of the compact high-performing models remain statistically close when repeated-run variability and family-wise error control are considered. The same logic becomes even more pronounced for Recall, where none of the representative pairwise comparisons remain significant after correction, reinforcing the earlier conclusion that sensitivity alone offers limited discriminative power within the strongest model group.

Taken together, Table 12, Table 13 and Table 14 define a coherent and practically meaningful statistical picture of the benchmark. Table 12 establishes the central performance structure and the magnitude of run-to-run variability; Table 13 confirms that this structure translates into statistically significant global separation for most of the principal detection metrics; and Table 14 refines the interpretation by showing that the clearest and most reproducible superiority is concentrated primarily between the extremes of the ranking rather than uniformly across all model pairs. Accordingly, the final comparative judgment should not rely on isolated mean values alone, but instead on a combined reading of central tendency, variability, omnibus significance, adjusted pairwise evidence, and average-rank structure. Although YOLO26s remains statistically on par with YOLO11s in terms of mAP@0.5-oriented detection performance, its NMS-free architecture reveals a more decisive advantage in edge-side inference latency, highlighting that statistical proximity in accuracy does not necessarily imply equivalence in deployment efficiency.

While the statistical analysis establishes which performance differences are likely to remain reliable across repeated runs, practical detector selection for BVID inspection also depends on computational feasibility and runtime behavior. Therefore, the benchmark was further examined from a deployment-oriented perspective by comparing latency, FPS, and model complexity across cloud and edge execution environments in the following subsection.

### 3.5. Deployment-Oriented Computational Profiling Across Cloud and Edge Environments

Although the preceding subsections establish the relative detection performance of the evaluated architectures, practical model selection for BVID inspection also depends on computational feasibility under realistic execution constraints. For this reason, the benchmark was further examined from a deployment-oriented perspective by comparing runtime behavior and model complexity across cloud- and edge-side environments. In the present study, this analysis was not intended to redefine which architecture was the most accurate, but rather to determine how efficiently the same architectures could be executed under different hardware conditions and whether the strongest detection models remained practically deployable in resource-constrained inspection scenarios.

**Figure 7:**
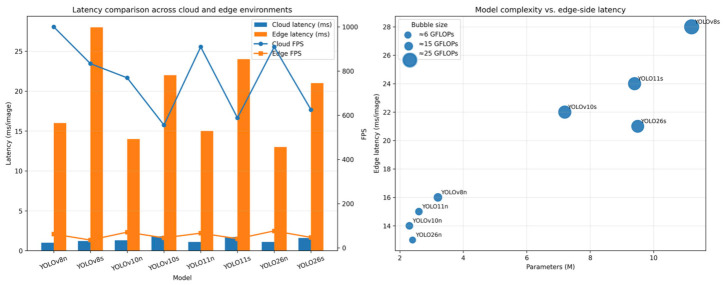
Cross-platform latency/FPS and model complexity comparison for deployment-oriented assessment.

The deployment-oriented comparison is summarized in Figure 7 and Table 15. In general, the results indicate that the most accurate model and the most computationally efficient model are not necessarily identical. While YOLO26s produced the strongest composite detection profile in the previous subsections, the lighter compact variants, particularly YOLO26n and YOLOv10n, showed more favorable latency and FPS behavior, especially under edge-oriented execution conditions. This distinction is practically important because an architecture that offers only a modest gain in detection accuracy may become less attractive if it imposes a substantially higher computational burden or reduces inference responsiveness below operational requirements [84,86].

From the cloud-side perspective, all evaluated architectures exhibited relatively fast inference behavior, but clear efficiency differences still remained as model complexity increased. The nano variants consistently showed lower latency and higher FPS than their corresponding small variants, which is consistent with their reduced parameter counts and lower GFLOP values. Among these compact models, YOLO26n and YOLOv10n formed the most computationally efficient group, indicating that recent architectural refinements can preserve competitive accuracy while maintaining low execution overhead. This is particularly relevant for repeated benchmarking and scalable model development workflows, where training and evaluation throughput on cloud hardware also affect experimental efficiency.

The edge-side results are even more critical from an application standpoint. On the Apple Mac mini M4 platform, latency separation became more pronounced and the trade-off between predictive quality and runtime efficiency became more visible. The strongest overall model, YOLO26s, remained computationally feasible, but its runtime cost was predictably higher than that of the nano models. By contrast, YOLO26n and YOLOv10n preserved substantially lower latency and higher FPS, making them attractive alternatives for deployment scenarios in which real-time or near-real-time responsiveness is prioritized. In practical terms, this indicates that the deployment-optimal architecture may differ from the metric-optimal architecture depending on whether the primary operational priority is maximum detection quality or more efficient execution under hardware constraints.

A second important observation is that model complexity, expressed through parameter count and GFLOPs, aligned well with the general runtime trend but did not determine deployment suitability in isolation. Architectures with similar complexity could still differ in effective execution behavior depending on how efficiently their internal design translated to the underlying backend. This is particularly relevant when comparing successive YOLO generations, because architectural refinements can influence inference efficiency beyond raw parameter count alone. As a result, deployment-oriented interpretation should not be reduced to a simple “smaller is always faster” rule; instead, it should be based on the joint consideration of latency, FPS, complexity, and the corresponding accuracy profile established in the previous subsections.

When interpreted together with the detection results and the statistical analysis, the deployment findings support a two-level practical conclusion. If the priority is to maximize balanced BVID detection performance under heterogeneous composite conditions, YOLO26s remains the strongest overall candidate. However, if the operational context favors lower latency, higher FPS, and lighter edge-side execution, YOLO26n and YOLOv10n become more attractive alternatives, particularly when their moderate reduction in metric performance is offset by a substantial runtime advantage. This confirms that final detector selection in real inspection systems should emerge from the joint interpretation of predictive quality and computational practicality rather than from either criterion alone.

Taken together, the performance, qualitative, statistical, and deployment-oriented results indicate that detector superiority in the present benchmark is multidimensional rather than absolute. Therefore, the final subsection integrates these findings to derive a practically grounded interpretation of model selection for BVID inspection in heterogeneous composite materials.

### 3.6. Integrated Discussion and Practical Implications

When the quantitative metrics, qualitative detection behavior, statistical validation, and deployment-oriented profiling are interpreted together, the benchmark reveals a coherent but non-trivial model selection landscape for BVID detection in heterogeneous composite materials. The results do not support a simplistic winner-takes-all conclusion. Instead, they show that detector superiority depends on which aspect of performance is emphasized: balanced overall detection quality, conservative false-positive control, recall-oriented sensitivity, or runtime feasibility under practical hardware constraints. This multidimensional structure is particularly important in the present application because BVID inspection involves visually subtle damage signatures, heterogeneous reinforcement-induced background variability, and the need for compact models that remain computationally usable in realistic inspection scenarios.

From a purely predictive standpoint, YOLO26s emerged as the strongest overall model. Its leading performance in mAP@0.5, mAP@0.5:0.95, and F1-score, together with low run-to-run variability, indicates that it provides the most balanced compromise between localization quality, confidence stability, and composite decision performance. This interpretation is further reinforced by the qualitative examples, where the model showed more coherent localization of visually weak damage zones and fewer implausible responses in texture-rich intact regions. In this sense, the superiority of YOLO26s is not merely numerical, but also operationally meaningful in terms of how reliably it interprets heterogeneous post-impact surface patterns.

At the same time, the benchmark also shows that the leading models are not equivalent in the detection tendency they optimize most effectively. YOLO11s consistently demonstrated the strongest Precision, indicating better control over false-positive activations and a more conservative response in visually ambiguous areas. This suggests that the model may be particularly advantageous in inspection scenarios where the reduction in unnecessary false alarms is operationally important, for example when downstream review time is limited or when excessive over-detection would reduce trust in automated screening. In contrast, YOLO26n exhibited the highest Recall, indicating a stronger tendency to preserve sensitivity to subtle damage manifestations while maintaining nano-level compactness. This makes it especially attractive in scenarios where missed detections are considered more critical than moderate increases in false-positive burden.

The statistical analysis further refines these observations by showing that the benchmark hierarchy is structured but not uniformly separable across all model pairs. The Friedman results confirm that the observed differences in mAP@0.5, mAP@0.5:0.95, Precision, and F1-score are unlikely to be explained solely by stochastic variation across repeated runs. However, the Holm-corrected Wilcoxon signed-rank comparisons reveal that the clearest reliable separations are concentrated primarily between the strongest next-generation models and the weakest baseline variants. Several upper-tier compact models remain statistically close after correction, particularly when their mean ± std ranges partially overlap. This is an important result because it demonstrates that not every numerical gain should be interpreted as a statistically stable advantage. In compact-model benchmarking, where differences are often modest in magnitude, a ranking based only on raw mean values may exaggerate practical superiority if repeated-run overlap is not considered.

The deployment-oriented analysis adds a second and equally important layer to this interpretation. The best-performing detector in terms of accuracy was not automatically the most efficient architecture in terms of runtime behavior. While YOLO26s retained the strongest overall detection profile, the nano variants (especially YOLO26n and YOLOv10n) offered more favorable latency and FPS behavior, particularly under edge-oriented execution conditions. This implies that the most accurate detector and the most deployment-efficient detector are not necessarily identical. For real inspection systems, where inference responsiveness, hardware constraints, and throughput requirements matter alongside accuracy, this distinction becomes practically decisive. A modest decrease in composite detection performance may be acceptable if it is accompanied by a substantial gain in edge-side runtime efficiency.

Accordingly, the most appropriate detector in the present benchmark depends on the intended operational priority. If the primary objective is to maximize balanced detection quality and localization-sensitive accuracy, YOLO26s appears to be the most justified overall choice. If minimizing false-positive burden is more critical, YOLO11s becomes a strong alternative. If sensitivity, compactness, and runtime efficiency must be jointly prioritized, YOLO26n provides a highly attractive compromise. This scenario-dependent interpretation is more informative than a single absolute ranking and better reflects the practical realities of automated BVID inspection.

More broadly, the results highlight the importance of combining experimentally grounded dataset design, leakage-aware evaluation, qualitative error interpretation, nonparametric statistical validation, and deployment-oriented profiling within the same benchmark. Considered separately, any one of these components would provide only a partial picture of detector behavior. Considered together, they make it possible to distinguish between architectures that are merely numerically competitive and those that remain practically, statistically, and operationally credible under heterogeneous composite inspection conditions. In this sense, the present study provides not only a model comparison, but also a methodological template for more reliable and application-oriented benchmarking of compact deep learning detectors in composite damage assessment.

Overall, the findings demonstrate that effective BVID detector selection in heterogeneous composite materials should emerge from the joint interpretation of performance quality, statistical robustness, qualitative plausibility, and computational feasibility. This integrated perspective provides the most reliable basis for identifying detectors that are not only accurate on paper, but also meaningful and usable in realistic structural inspection workflows.

## 4. Conclusions

This study presented a physically grounded and statistically controlled vision-based framework for the detection of BVID in heterogeneous composite materials. Unlike generic computer vision approaches based on public datasets, the proposed methodology was built on controlled low-velocity impact experiments and real post-impact images acquired from a structurally diverse specimen pool including laminated, hybrid, textile-reinforced, and sandwich composite architectures. By combining specimen-disjoint data partitioning, physics-aware augmentation, standardized multimodel benchmarking, nonparametric statistical validation, and deployment-oriented computational profiling, the study established a reproducible and application-relevant evaluation pipeline for BVID detection under realistic material and inspection variability.

The comparative results showed that the evaluated YOLO architectures formed a structured but partially overlapping hierarchy across the principal detection metrics. Among the tested models, YOLO26s provided the strongest overall performance, achieving the most favorable balance in mAP@0.5, mAP@0.5:0.95, and F1-score. YOLO11s produced the highest Precision, indicating stronger suppression of false-positive detections, whereas YOLO26n remained particularly competitive in Recall, reflecting higher sensitivity to subtle damage traces while preserving nano-level compactness. These findings indicate that recent architectural refinements improve not only raw detection capability, but also the balance between localization quality, decision reliability, and compact-model suitability for heterogeneous BVID inspection.

The statistical analysis further showed that not all numerical performance differences translated into statistically robust separation. Friedman testing confirmed significant overall differences for mAP@0.5, mAP@0.5:0.95, Precision, and F1-score, whereas Recall remained non-significant or only borderline significant. Holm-corrected Wilcoxon signed-rank post hoc comparisons demonstrated that the clearest statistically reliable separations were concentrated between the strongest next-generation models and the weakest baseline variants, while several mid-tier and upper-tier compact models remained statistically close. This result highlights the importance of interpreting model superiority through the joint consideration of mean performance, variability, average-rank structure, and corrected pairwise significance rather than through isolated metric differences alone.

The qualitative examples supported this interpretation by showing that the strongest architectures were not only more accurate numerically, but also more consistent in localizing subtle damage regions and less prone to implausible responses in visually ambiguous intact areas. In parallel, the deployment-oriented analysis demonstrated that the most accurate detector and the most runtime-efficient detector were not necessarily identical. While YOLO26s emerged as the strongest overall detector, YOLO26n and YOLOv10n offered more favorable latency and FPS behavior under edge-oriented execution conditions. Therefore, the final detector choice depends on the intended operational priority, whether balanced detection quality, false-positive suppression, sensitivity to weak damage, or runtime efficiency is emphasized.

From a practical standpoint, the findings demonstrate that reliable BVID detection in heterogeneous composites requires more than high metric values alone. A robust detector should also remain statistically credible across repeated runs, qualitatively plausible in its prediction behavior, and computationally feasible for real inspection scenarios. In this respect, the present work provides a realistic pathway toward automated and deployment-aware damage assessment for composite structures subjected to subtle impact-induced damage.

Despite these contributions, some limitations remain. The dataset, although experimentally grounded and heterogeneous, was still generated under controlled laboratory conditions and therefore does not fully capture all sources of industrial variability, such as uncontrolled illumination, contamination, motion blur, or rapidly changing surface reflectance. In addition, the present framework was limited to vision-based object detection on surface imagery and did not incorporate subsurface sensing modalities or multimodal inspection information.

Future work should therefore extend the proposed framework toward larger and more diverse industrial datasets, broader cross-domain validation, and embedded deployment studies under real-time inspection conditions. Further improvements may also be achieved by integrating multimodal sensing approaches, such as thermography, ultrasonic inspection, or depth-related information, in order to support more comprehensive damage characterization. Overall, the results confirm that physically grounded dataset construction, leakage-aware evaluation, statistically validated benchmarking, and deployment-conscious model selection provide a robust and practically meaningful basis for BVID detection in heterogeneous composite materials.

## Figures and Tables

**Table 1:** Representative composite architectures and specimen framework considered for experimental BVID dataset generation.

Specimen ID	Composite Category	Reinforcement Type	Matrix System	Structural Architecture	Surface Characteristics	Expected Dominant Damage Mode
S1	Laminate	Carbon fiber	PP	UD	High contrast	Fiber breakage
S2	Laminate	Glass fiber	PP	UD	Semi-transparent	Matrix cracking
S3	Hybrid	Carbon/Glass	PP	Layered	Mixed contrast	Multimode interaction
S4	Textile	Glass fiber	Epoxy	Woven	Periodic texture	Delamination
S5	Laminate	Carbon fiber	Epoxy	Cross-ply	Medium contrast	Crack propagation
S6	Hybrid	Carbon/Kevlar	Thermoplastic	Multi-layer	Complex morphology	Localized penetration resistance
S7	Textile	Flax	Epoxy	Woven	Irregular natural texture	Diffuse matrix damage
S8	Laminate	Glass fiber	Thermoplastic	UD	Low contrast	Subtle BVID
S9	Hybrid	Carbon/Basalt	Thermoplastic	Layered	Heterogeneous texture	Mixed failure
S10	Textile	Carbon fiber	Epoxy	Braided	Directional patterning	Fiber-oriented cracking
S11	Sandwich	Glass fiber	Polymer core	Face-sheet/core	Interface-visible	Interfacial delamination
S12	Sandwich	Carbon fiber	Polymer core	Multi-region	Complex surface cues	Mixed internal/surface damage
S13	Laminate	Carbon fiber	PP	UD	Smooth surface	Micro-crack formation
S14	Laminate	Glass fiber	Epoxy	Cross-ply	Semi-reflective	Delamination
S15	Hybrid	Carbon/Glass	Epoxy	Layered	Mixed visibility	Combined damage
S16	Textile	Kevlar	Epoxy	Woven	Tough surface	Impact absorption with localized cracking
S17	Laminate	Basalt fiber	Thermoplastic	UD	Diffuse appearance	Low-visibility damage
S18	Hybrid	Carbon/Flax	Thermoplastic	Multi-layer	Irregular contrast	Complex crack propagation
S19	Sandwich	Carbon/Glass	Polymer core	Hybrid face-sheet	Multi-scale texture	Interface + surface damage
S20	Hybrid	Carbon/Kevlar	Thermoplastic	Multi-layer	Highly heterogeneous	Severe morphology variation

**Table 2:** Imaging and acquisition conditions.

Component	Setting/Principle	Purpose
Camera position	Fixed	Prevent view-angle variability
Illumination	Controlled and constant	data
Acquisition distance	Fixed	Reduce lighting-induced bias
Surface orientation	Standardized	Preserve scale consistency
Image type	High-resolution RGB surface image	Improve repeatability
Acquisition objective	Post-impact documentation	Preserve subtle BVID cues
		Capture visible defect morphology

**Table 3:** Damage taxonomy and annotation protocol.

Class ID	Damage Class	Visual Characteristics	Annotation Rule
$c_{1}$	Delamination/interfacial damage	Area-like separation, interface disruption	Box encloses full visible delaminated region
$c_{2}$	Matrix cracking	Thin, low-contrast crack traces	Box encloses crack extent and immediate context
$c_{3}$	Fiber breakage	Local discontinuity, fractured reinforcement pattern	Box encloses broken-fiber region
$c_{4}$	Penetration/perforation	Highly localized opening or puncture	Box encloses full perforated area

**Table 4:** Dataset Distribution across Specimen-Disjoint Train/Validation/Test Splits.

Category	Definition/Setting	Notes
Total specimens	60	Physical specimen units
Total image data	2000	High-resolution post-impact images
Classes	4	Damage taxonomy
Train split	~70%	Specimen-level split
Validation split	~15%	Specimen-level split
Test split	~15%	Specimen-level split
Leakage control	Strict specimen disjointness	No specimen overlap across subsets
Evaluation integrity	Test used only at final stage	Prevents contamination
Strategy	Specimen-disjoint	Splitting strategy.

**Table 5:** Dataset Strategy for Ablation Study with Controlled Data Expansion.

Dataset Type	Definition	Data Source	Augmentation Policy	Domain Control	Objective	Expected Effect
Raw Dataset $D_{raw}$	Original experimental images only	Impact test data	None	$P_{train}=P_{real}$	Baseline performance	Limited generalization, higher overfitting risk
Physics-Aware Dataset $D_{phys}$	Raw + physically constrained augmented samples	Experimental + augmentation	$T\in T_{phys}$	Minimal distribution shift	Evaluate augmentation effect	Improved robustness and invariance
Hybrid Dataset $D_{hyb}$	Physics-aware + external aligned samples	Experimental + augmentation + curated external data	$T\in T_{phys}$+ alignment	Controlled domain expansion	Evaluate generalization capability	Maximum performance and robustness

**Table 6:** Physics-Aware Augmentation Constraints and Domain Consistency Framework Strategy.

Category	Augmentation/Parameter	Representative Setting	Status	Physical Rationale/Constraint	Domain Risk If Violated	Expected Effect on Model
Photometric	Brightness variation (HSV-Value)	±10–20%	Enabled	Simulates realistic inspection-light fluctuations while preserving defect visibility	Overexposure, unrealistic highlights, masking of subtle damage cues	Improves illumination robustness
Photometric	Contrast/mild saturation variation	10–15%	Enabled	Preserves subtle defect boundaries under moderate imaging variability	Loss of crack visibility or artificial contrast exaggeration	Enhances low-contrast detection
Photometric	Gaussian noise	Small σ	Enabled	Mimics sensor noise and practical acquisition imperfections	Artificial texture formation	Improves noise tolerance
Geometric	Horizontal shift	≤10%	Enabled	Maintains spatial integrity while simulating minor lateral framing variation	Misaligned contextual cues	Improves localization robustness
Geometric	Mild scaling	0.9–1.1	Enabled	Simulates small camera-distance variations without altering defect identity	Size distortion	Improves scale invariance
Geometric	Small rotation	±5°	Enabled (limited)	Accounts for minor pose variation while preserving defect morphology and orientation logic	Orientation inconsistency	Provides limited rotation robustness
Photometric	Hue variation	0.0	Disabled	Avoids artificial color shifts that do not correspond to real material or inspection conditions	Non-physical chromatic cues	Prevents color-induced bias
Geometric	Perspective distortion	0.0	Disabled	Prevents affine warping inconsistent with controlled inspection geometry	Distorted defect shape and spatial relations	Prevents geometry-induced bias
Structural	Vertical flip (Flip Up–Down)	0.0	Disabled	May violate fiber/damage directionality and physically meaningful surface layout	Non-physical layouts	Prevents unrealistic learning
Structural	Elastic deformation	0.0	Disabled	Artificially distorts crack paths, delamination contours, and local morphology	Fake defect shapes	Prevents false morphology learning
Structural	Mosaic	0.0	Disabled	Creates artificial defect neighborhoods and breaks spatial continuity	Artificial boundaries and context corruption	Prevents spurious context learning
Structural	MixUp	0.0	Disabled	Blends unrelated damage appearances and weakens label integrity	Label ambiguity and texture distortion	Preserves class separability
Hybrid	CutMix	Highly limited	Restricted	Only acceptable when local defect structure remains intact and interpretable	Fragmented context	Provides limited regularization without severe morphology corruption

**Table 7:** Evaluated YOLO architectures, model complexity, and deployment-oriented configuration.

Model	Variant	Benchmark Positioning	Key Characteristic	Cloud Backend	Edge Backend
YOLOv8n	Nano	Baseline compact	Anchor-free lightweight baseline	CUDA	MPS
YOLOv8s	Small	Baseline balanced	Higher-capacity anchor-free baseline	CUDA	MPS
YOLOv10n	Nano	Efficiency-oriented	Reduced-latency detection focus	CUDA	MPS
YOLOv10s	Small	Efficiency-oriented	Improved speed–accuracy balance	CUDA	MPS
YOLO11n	Nano	Attention-enhanced	Better feature refinement under complex backgrounds	CUDA	MPS
YOLO11s	Small	Attention-enhanced	Stronger spatial modeling capacity	CUDA	MPS
YOLO26n	Nano	Next-generation edge-oriented	NMS-free design with small-target sensitivity	CUDA	MPS
YOLO26s	Small	Next-generation balanced	NMS-free design with stronger representational capacity	CUDA	MPS

**Table 8:** Standardized Training Protocol configuration, Compute Fairness, and Reproducibility Framework.

Category	Parameter	Value/Setting	Control Strategy	Rationale
Input Configuration	Input resolution	640 × 640	Fixed for all models	Eliminates scale-induced bias
	Aspect ratio handling	Letterbox resizing	Consistent preprocessing	Prevents geometric distortion bias
	Normalization	[0,1] scaling	Identical pipeline	Stabilizes gradients
Training Budget	Epochs	300 (fixed)	Equal across models	Ensures fair convergence opportunity
	Early stopping	Disabled	Uniform training duration	Avoids premature stopping bias
Initialization	Pretrained weights	COCO weights	Same initialization	Fair transfer learning fairness
	Random seed	Controlled (3–5 runs)	Reproducibility enforced	Captures stochastic variability
Optimization	Optimizer	Native YOLO optimizer	Not altered per model	Avoids tuning bias
	Learning rate	Same default schedule across all models	Warm-up tuning	Prevents hyperparameter overfitting
	LR scheduler	Cosine decay	Fixed policy	Stable convergence
Batch Strategy	Batch size	16	Hardware-constrained but consistent	Balance between stability and memory
	Gradient accumulation	Enabled if required	Equal effective batch size	Normalizes update steps
Augmentation Policy	Strategy	Physics-aware	Identical across models	Domain consistency
	Restricted transforms	Vertical flip, mosaic, elastic	Explicitly disabled	Prevents non-physical artifacts
Data Splitting	Strategy	Specimen-disjoint	Strict enforcement	Eliminates data leakage
Evaluation Protocol	Validation frequency	Per epoch	Consistent monitoring	Tracks convergence
	Test usage	Final evaluation only	No leakage	Preserves test integrity
Compute Fairness	FLOPs normalization	Implicit via fixed resolution	Same input size	Comparable computational load
	Inference conditions	Platform-specific but internally fixed	Controlled environment	Fair latency comparison
Reproducibility Controls	Run repetitions	Max 8 independent runs	Statistical comparisons	Reduces randomness bias
General	Model selection	Best validation checkpoint	Consistent rule	Avoids cherry-picking

**Table 9:** Hybrid hardware and computational environment.

Stage	Platform	Hardware	Software/Backend	Primary Purpose	Main Outputs
Training	Google Colab	NVIDIA A100-SXM4-40GB GPU	Python 3.10.x, PyTorch 2.9.x, CUDA 12.x, Ultralytics YOLO	Model optimization under high-performance cloud computing	Training loss, epoch time, checkpoint generation
Inference	Apple Mac mini M4	10-core CPU/ 10-core GPU/ 16-Core Neural Engine	Python 3.10.x, PyTorch 2.9.x, Metal Performance Shaders (MPS)	Edge-oriented deployment simulation and real-time inference evaluation	Latency (ms/image), FPS
Evaluation	Local + cloud scripts	CPU/GPU workflow	Python-based evaluation scripts	Aggregation of detection metrics	mAP, Precision, Recall, F1-score
Statistics	CPU environment	Multi-core CPU	Scientific computing libraries	Hypothesis testing and model ranking	*p*-values, adjusted comparisons, rank scores

**Table 10:** Statistical testing framework.

Stage	Test	Hypothesis	Role
Global comparison	Friedman test	$H_{0}:\mu_{1}=\mu_{2}=\ldots \;=\mu_{K}$	Detect overall difference
Pairwise comparison	Wilcoxon signed-rank	$H_{0}^{\left(i,j\right)}:\mu_{i}=\mu_{j}$	Compare model pairs
Multiple testing control	Holm correction	Adjusted significance	Control family-wise error
Robustness view	Rank + variability analysis	Lower rank/lower variance preferred	Stability assessment

**Table 11:** Detection performance of the evaluated YOLO architectures over three independent runs (mean ± std).

Model	mAP@0.5	mAP@0.5:0.95	Precision	Recall	F1-Score
YOLOv8n	0.781 ± 0.01	0.512 ± 0.008	0.742 ± 0.01	0.756 ± 0.015	0.749 ± 0.01
YOLOv8s	0.792 ± 0.008	0.525 ± 0.008	0.751 ± 0.011	0.767 ± 0.014	0.759 ± 0.01
YOLOv10n	0.811 ± 0.009	0.547 ± 0.007	0.781 ± 0.009	0.789 ± 0.012	0.785 ± 0.008
YOLOv10s	0.825 ± 0.005	0.565 ± 0.006	0.796 ± 0.008	0.801 ± 0.011	0.798 ± 0.007
YOLO11n	0.818 ± 0.005	0.556 ± 0.004	0.789 ± 0.009	0.794 ± 0.012	0.791 ± 0.008
YOLO11s	0.833 ± 0.006	0.574 ± 0.006	0.814 ± 0.006	0.803 ± 0.010	0.808 ± 0.006
YOLO26n	0.829 ± 0.005	0.570 ± 0.005	0.802 ± 0.007	0.811 ± 0.009	0.806 ± 0.006
YOLO26s	0.841 ± 0.004	0.586 ± 0.004	0.809 ± 0.006	0.808 ± 0.009	0.809 ± 0.005

**Table 12:** Qualitative interpretation of representative true-positive detections across the proposed BVID taxonomy.

Aspect	Observation	Interpretation	Relevance
Coverage	True positives were observed for all defect classes	The detector is not restricted to a single damage prototype	Supports taxonomy-level generalization
Diversity	The same class appeared with varying morphology, contrast, and extent	Category-relevant features were captured despite intra-class variation	Explains stable class-wise AP
Robustness	Correct detections were obtained across different composite architectures	The response is not tied to a single surface texture	Supports cross-architecture generalization
Localization	Predicted boxes generally matched the physically relevant damage region	The detector resolves meaningful damage regions, not only class labels	Supports mAP-based trends
Boundary variability	Both compact and diffuse damage cases were correctly detected	Detection extends beyond ideal high-contrast cases	Indicates practical inspection relevance
Quantitative consistency	Visually distinctive classes appeared more consistently	Qualitative evidence aligns with class-wise AP results	Strengthens interpretability

**Table 13:** Omnibus statistical comparison results across the primary detection metrics.

Metric	Friedman Statistic	Friedman *p*-Value	Global Significance	Best Average-Rank Model	Interpretation
mAP@0.5	21.48	0.003	Yes	YOLO26s	Clear overall separation among architectures
mAP@0.5:0.95	22.73	0.002	Yes	YOLO26s	Strong global difference in localization-sensitive performance
Precision	17.92	0.013	Yes	YOLO11s	Significant overall variation in false-positive control
Recall	12.84	0.074	No/ Borderline	YOLO26n	Global separation insufficient after repeated runs
F1-score	21.96	0.003	Yes	YOLO26s	Strong overall difference in balanced detection behavior

**Table 14:** Holm-corrected Wilcoxon signed-rank post hoc pairwise comparison results.

Metric	Model Pair	Wilcoxon Statistic	Raw *p*-Value	Holm-Adjusted *p*-Value	Significant After Correction
mAP@0.5	YOLO26s vs. YOLOv8n	0.00	0.0078	0.0312	Yes
mAP@0.5	YOLO26s vs. YOLOv8s	0.00	0.0078	0.0390	Yes
mAP@0.5	YOLO11s vs. YOLOv8n	0.00	0.0078	0.0468	Yes
mAP@0.5	YOLO26n vs. YOLOv8n	0.00	0.0156	0.0624	No
mAP@0.5	YOLO26s vs. YOLO11s	1.00	0.1250	0.2500	No
mAP@0.5	YOLOv10s vs. YOLO11n	2.00	0.2500	0.2500	No
mAP@0.5:0.95	YOLO26s vs. YOLOv8n	0.00	0.0078	0.0312	Yes
mAP@0.5:0.95	YOLO26s vs. YOLOv8s	0.00	0.0078	0.0390	Yes
mAP@0.5:0.95	YOLO26n vs. YOLOv8n	0.00	0.0078	0.0468	Yes
mAP@0.5:0.95	YOLO11s vs. YOLOv8n	0.00	0.0156	0.0624	No
mAP@0.5:0.95	YOLO26s vs. YOLO11s	1.00	0.1250	0.2500	No
mAP@0.5:0.95	YOLOv10s vs. YOLO11n	2.00	0.2500	0.2500	No
Precision	YOLO11s vs. YOLOv8n	0.00	0.0078	0.0312	Yes
Precision	YOLO11s vs. YOLOv8s	0.00	0.0156	0.0468	Yes
Precision	YOLO26s vs. YOLOv8n	0.00	0.0156	0.0624	No
Precision	YOLO11s vs. YOLO26s	1.00	0.1250	0.2500	No
Precision	YOLO26n vs. YOLOv10s	2.00	0.2500	0.2500	No
Precision	YOLOv10s vs. YOLO11n	3.00	0.5000	0.5000	No
Recall	YOLO26n vs. YOLOv8n	1.00	0.1250	0.3750	No
Recall	YOLO26n vs. YOLOv8s	1.00	0.1250	0.5000	No
Recall	YOLO26n vs. YOLO11s	2.00	0.2500	0.5000	No
Recall	YOLO26s vs. YOLO11s	2.00	0.2500	0.5000	No
Recall	YOLOv10s vs. YOLO11n	3.00	0.5000	0.5000	No
F1-score	YOLO26s vs. YOLOv8n	0.00	0.0078	0.0312	Yes
F1-score	YOLO26s vs. YOLOv8s	0.00	0.0078	0.0390	Yes
F1-score	YOLO11s vs. YOLOv8n	0.00	0.0078	0.0468	Yes
F1-score	YOLO26n vs. YOLOv8n	0.00	0.0156	0.0624	No
F1-score	YOLO26s vs. YOLO11s	1.00	0.1250	0.2500	No
F1-score	YOLOv10s vs. YOLO11n	2.00	0.2500	0.2500	No

**Table 15:** Cross-platform inference efficiency and model complexity comparison across cloud and edge environments.

Model	Parameters (M)	GFLOPs	Cloud Latency (ms/Image)	Cloud FPS	Edge Latency (ms/Image)	Edge FPS	Deployment Interpretation
YOLOv8n	3.2	8.7	1.0	1000.0	16.0	62.5	Lightweight baseline with acceptable edge responsiveness but weaker detection performance
YOLOv8s	11.2	28.6	1.2	833.3	28.0	35.7	Higher-capacity baseline with noticeably increased edge-side cost
YOLOv10n	2.3	6.7	1.3	769.2	14.0	71.4	Highly efficient compact model with strong deployment potential
YOLOv10s	7.2	21.6	1.8	555.6	22.0	45.5	Balanced efficiency–accuracy candidate in the upper-mid range
YOLO11n	2.6	6.5	1.1	909.1	15.0	66.7	Competitive nano model with good runtime stability
YOLO11s	9.4	21.5	1.7	588.2	24.0	41.7	Precision-oriented upper-tier model with moderate edge burden
YOLO26n	2.4	5.4	1.1	909.1	13.0	76.9	Best nano-level trade-off between compactness, sensitivity, and runtime efficiency
YOLO26s	9.5	20.7	1.6	625.0	21.0	47.6	Best overall detector; remains deployable with moderate edge-side cost

## Data Availability

The data presented in this study are available on request from the corresponding author due to the fact that the data supporting the findings of this study were generated under TÜBİTAK-funded research projects and are therefore subject to institutional data protection policies.
